# Sirtuin 7: a new marker of aggressiveness in prostate cancer

**DOI:** 10.18632/oncotarget.20468

**Published:** 2017-08-24

**Authors:** Romain Haider, Fabienne Massa, Lisa Kaminski, Stephan Clavel, Zied Djabari, Guillaume Robert, Kathiane Laurent, Jean-François Michiels, Matthieu Durand, Jean-Ehrland Ricci, Jean-François Tanti, Frédéric Bost, Damien Ambrosetti

**Affiliations:** ^1^ Université Côte d’Azur, C3M-Inserm U1065, Nice, France; ^2^ Urology Department, CHU Nice, Nice, France; ^3^ Pathology Department, CHU Nice, Nice, France

**Keywords:** prostate cancer, sirtuins, metastasis, metabolism, cell migration

## Abstract

Predictive biomarkers for advanced prostate cancer (PCa) are still missing. The sirtuin 7 (SIRT7) has been linked to tumorogenesis but its role in prostate cancer is poorly documented. To determine if SIRT7 can be a biomarker for aggressive prostate cancer and plays a role in PCa aggressiveness. We analyzed the expression of SIRT7 by immunohistochemistry in 57 patients comparing healthy with adjacent cancer tissue. SIRT7 levels were significantly elevated in tumors and its expression was positively associated with the grade. We also demonstrated that the knock down of SIRT7 decreased the migration of DU145 and PC3 cells (two androgen-independent prostate cancer cell lines) whereas the overexpression of the native protein but not the mutated form increased the cell migration and the invasion of the poorly aggressive prostate cancer cell line LNCaP. Finally, we also showed that SIRT7 overexpression induced the resistance to docetaxel. Our results demonstrate that SIRT7 promotes prostate cancer cell aggressiveness and chemoresistance and suggest that SIRT7 is a good predictive biomarker of PCa aggressiveness.

## INTRODUCTION

Prostate cancer (PCa) is one of the leading cause of death by cancer in men. Although effective surgical and radiation treatments exist for localized PCa, metastatic PCa remains mostly incurable. A major issue is to predict whether localized PCa will become metastatic, thus, markers of aggressiveness in PCa are needed. The process of metastasis is complex, it involves multiple biological processes, including angiogenesis, local migration and invasion, of tumor cells [1]. PCa cell migration and invasion are regulated by numerous proteins including the transcription factor Slug/snail, the glycoprotein fibronectin (FN) and the intermediate filament Vimentin [2]. For instance, fibronectin has been shown to induce the expression of the matrix metalloproteinases expression 2 [3] and blocking FN with anti-FN antibodies resulted in a significant decrease in adhesion of LNCaP prostate cancer cells [4].

Sirtuin 7 (SIRT7) belongs to the sirtuin family. It is a highly conserved protein family with seven members in mammals (SIRT 1-7). Sirtuins are NAD^+^ dependent histone deacetylase and also, for some of them, NAD^+^-dependent ADP ribosyltransferases. SIRT7 is the latest discovered sirtuin and its function remains poorly understood. SIRT7 deficiency in mice leads to a lifespan decrease, cardiac hypertrophy [5], hepatic steatosis [6, 7], and deafness [8]. Among the known substrates of SIRT7 are PAF53, NPM1, GABP-β1, but more importantly, SIRT7 catalyzes the deacetylation of lysine 18 on histone H3 (H3K18), a marker of cancer aggressiveness and poor prognosis [9]. It has been shown that the deacetylation of H3K18 by repressing a tumor suppressive program maintains the transformed state of cancer [10]. Consequently, the downregulation of SIRT7 noticeably reduces tumor growth in mice models and inhibits the properties of cancer cells such as anchorage-independent growth [10].

In this article, we studied whether SIRT7 has a clinical relevance in PCa progression. We analyzed SIRT7 expression in PCa and we studied the role of the protein in PCa aggressiveness.

## RESULTS

### SIRT7 protein expression is higher in PCa

**Figure 1:**
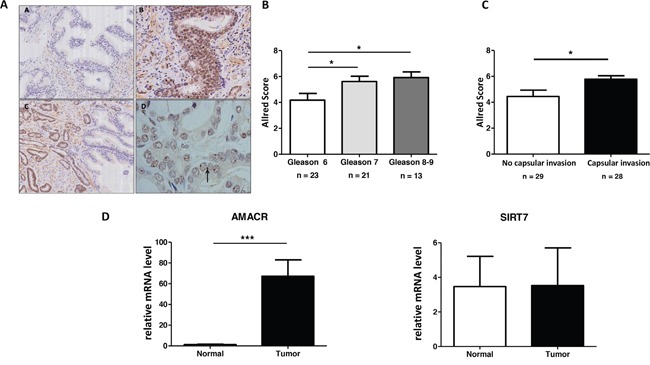
SIRT7 is overexpressed in PCa **(A)** Sirtuin 7 immunohistochemical staining on human prostate tissue. (A.a) Healthy prostate, absence of staining. (A.b) Prostate adenocarcinoma, nuclear staining of tumor cells. (A.c) On the edge of the tumor, no staining of healthy glands, nuclear staining of tumor cells. (A.d) Nucleolar staining of anti-SIRT7 antibody within a tumor area (arrow). **(B)** Association between All red Score and Gleason Score of index lesion in each surgical specimen. **(C)** Analysis of index lesion All red Score distribution according to the absence or the presence of prostatic capsular invasion in each surgical specimen. **(D)** Analysis of SIRT7 and AMACR mRNA expression in prostatic tumor cells.

A total of 57 surgical specimens of patients were included. Immunohistochemistry analysis on paraffin was achievable in 100% of cases. Clinico-pathological data are detailed in Table 1. Mean age of patients were 66.4 (+/-5.1) years old and mean PSA were 10.6 (+/-8.2) ng/ml. We reported 23, 21 and 13 patients with index lesions of Gleason Score 6, 7 and ≥8, respectively. SIRT7 immunohistochemical analysis evaluation is detailed in the Figure 1 and Supplementary Figure 1. There is no SIRT7 expression in healthy prostate tissue (Figure 1A. panel A) while the expression of SIRT7 was detected in tumor cells as illustrated by a nuclear staining of tumor cells in the prostate adenocarcinoma area (Figure 1A panel B). On the edge of the tumor, healthy glands are not stained and the nucleolus of tumor cells are stained (Figure 1A panel C). Of note, there is a clear nucleolar reinforcement of SIRT7 in some tumor regions independently of the grade (black arrow in Figure 1A panel D). The correlation between Allred Score and Gleason Score of index lesion in each surgical specimen was analyzed. An association was found between SIRT7 expression and tumor grade with a higher mean Allred Score in the Gleason Score 7 tumors (*p = 0.035*) and Gleason Score ≥ 8 (*p = 0.015*) compared with Gleason Score 6 tumors (Figure 1B). No statistically significant differences in Allred Score was found between Gleason Score 7 and > 8. The analysis of the index lesion Allred Score distribution according to the absence (0) or the presence (1) of prostatic capsular invasion in each surgical specimen revealed that the mean Allred Score was higher when there was a prostatic capsular invasion (*p = 0.02*) (Figure 1C).

**Table 1:** Patients characteristics of the 57 patients with advanced PCa

	n (%)
**Patients**	**57**
**Age (yo)**	
** median**	68 [51–74]
** mean**	66.4 (+/-5.1)
**PSA (ng/ml)**	
** median**	7.9 [3.2-39]
** mean**	10.6 (+/-8.2)
**D’Amico Score**	
** Lowrisk**	19 (33.3)
** Intermediaterisk**	18 (31.6)
** High risk**	20 (35.1)
**Tumor stage**	
** pT2a**	3 (5.3)
** pT2b**	1 (1.7)
** pT2c**	26 (45.6)
** pT3a**	22 (38.6)
** pT3b**	5 (8.8)
**Gleason Score of Index Lesion**	
** Gleason 6**	23 (40.3)
** Gleason 7**	21 (36.9)
** Gleason ≥ 8**	13 (22.8)

We extracted total mRNA from paraffin embedded tissues and performed a RT-qPCR analysis. The mRNA expression of SIRT7 and AMACR (an enzyme strongly expressed in PCa) are presented in Figure 1D. As expected, we found an up-regulation of AMACR expression in the tumor compared to the healthy gland whereas the expression of SIRT7 mRNA did not vary. This result suggests that the increase in SIRT7 expression in prostate cancer detected by immunohistochemical analysis could be due to a post transcriptional regulation.

Altogether, we demonstrated that the protein level of SIRT7 is significantly higher in PCa and it positively correlated with the grade of the lesion.

### The knockdown of SIRT7 decreased prostate cancer cells migration

**Figure 2:**
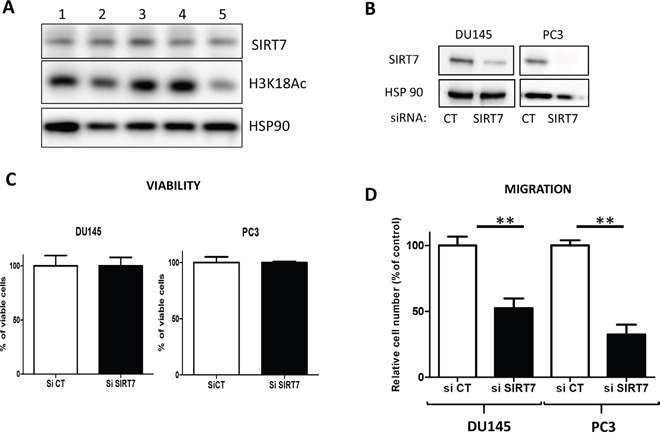
The knockdown of SIRT7 inhibits DU145 cell migration **(A)** Western blot analysis of SIRT7 and acetylated H3K18 in LNCaP (1), P69 (2), 22RV (3), DU145 (4) and PC3 (5) cells. **(B)** Western blot was performed to analyze SIRT7 expression in DU145 and PC3 cells transfected with siRNA. **(C)** Cell viability of DU145 and PC3 cells transfected with control SiRNA (SiCT) and SIRT7 siRNA (siSIRT7). The data represents three independent experiments with sem **(D)** Migration assay: the graph represents the number of cells/mm^2^ migrating across the boyden chamber in three independent experiments performed in triplicate as described in the materials and methods. (Student t-Test ** *p*<0.01).

To determine the role of SIRT7 in PCa, we analyzed the expression of SIRT7 and the acetylation of H3K18 in PCa cell lines (two androgen-dependent cell lines: LNCaP and 22RV and two androgen-independent cells lines DU145 and PC3) and one normal prostate epithelial cell line P69. The five cell lines have the same level of expression of SIRT7 and H3K18 is lower in PC3, the more aggressive cell line (Figure 2A). To study the potential role of SIRT7 in prostate cancer cell viability and aggressiveness, we downregulated the expression of SIRT7 in DU145 and PC3 cells, using two siRNA targeting two different regions of SIRT7 (Figure 2B and Supplementary Figure 2A). The depletion of SIRT7 did not affect the viability of the cells (Figure 2C) but it significantly inhibited the migration of both cell lines (Figure 2D and Supplementary Figure 2B). Indeed, the knockdown of SIRT7 led to the inhibition of the migration by 48% and 68 % in DU145 and PC3 respectively, suggesting that SIRT7 was implicated in prostate cancer cell migration and may participate in PCa aggressive phenotype.

### Overexpression of SIRT7 increased prostate cancer cell aggressiveness

**Figure 3:**
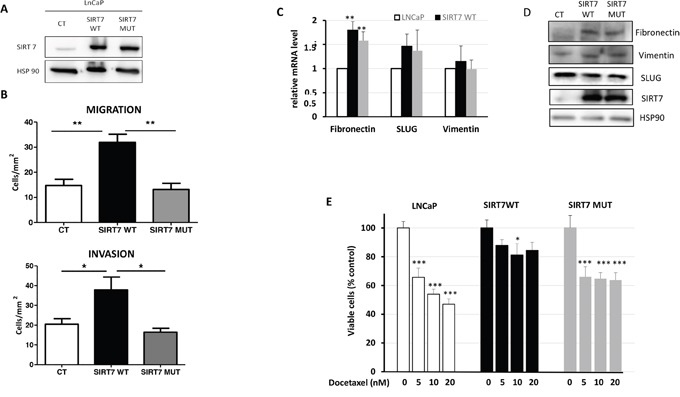
The overexpression of SIRT7 promotes LNCaP aggressiveness and chemoresistance **(A)** Western blot representing the expression of SIRT7 in the different clones: Control cells (CT), cells expressing SIRT7wt and SIRT7mut. **(B)** The graphs represent the number of cell/mm2 migrating across the boyden chamber in a migration and invasion assay. Three independent experiments were performed in triplicate as described in the materials and methods. **(C)** q-PCR of the expression of fibronectin, Slug and vimentin in the different populations of cells. The graph represents the results of five independent experiments. **(D)** Western blot showing the expression of fibronectin, vimentin, Slug, SIRT7 and HSP90 in SIRT7wt and SIRT7mut. **(E)** Viability assay in the different clones treated for 72h with the indicated concentrations of docetaxel. The graph represents the mean of four independent experiments performed in quadruplicate. (Student t-Test; * *p*<0.05; ** *p*<0.01; *** *p*<0.001).

To determine if there is a direct relationship between the level of SIRT7 and PCa cell aggressiveness, we overexpressed SIRT7 in the poorly aggressive, androgen-dependent human prostate cancer cell line LNCaP. We generated stable cell lines expressing wild-type SIRT7 (SIRT7wt) or a mutated form of SIRT7 (SIRT7mut) lacking the deacetylase activity of the sirtuin (Figure 3A). The constitutive expression of SIRT7wt and SIRT7mut did not affect clonogenic growth in a colony formation assay nor cell viability (Supplementary Figure 3A, 3B). In contrast, overexpression of SIRT7wt led to a significant increase in cell migration and invasion by 54% and 45% respectively (Figure 3B). Interestingly, the expression of the mutated form restored the wild-type phenotype, suggesting that the deacetylase activity of SIRT7 is implicated in the aggressiveness of prostate cancer cells. We then analyzed the expression of markers of the Epithelial Mesenchymal Transition (EMT). We showed that the expression of slug/snail and vimentin do not change in cell lines expressing SIRT7wt and SIRT7mut. On the contrary, we observed an increase of fibronectin mRNA and protein levels (Figure 3C, 3D).

Aggressiveness is associated with resistance to chemotherapy, thus, we assessed the implication of SIRT7 in the sensitivity to Docetaxel, the major chemotherapeutic agent for the treatment of prostate cancer. Increasing concentration of Docetaxel (5, 10 and 20nM) significantly decreased cell viability of the control cells and of the cells expressing SIRT7mut after 72h of treatment (Figure 3E). In contrast, it had no significant impact on the viability of SIRT7wt cells, suggesting that SIRT7 may be implicated in the resistance to Docetaxel. In conclusion, our data demonstrated that SIRT7 overexpression through its deacetylase activity confers aggressiveness and resistance to chemotherapy in LNCaP cells.

## DISCUSSION

We have demonstrated that SIRT7 plays an important role in the aggressiveness of prostate cancer. Our conclusion is based on clinical and experimental data. Firstly, the level of SIRT7 is significantly higher in PCa tumors and expressionist level is correlated with the grade of the tumor. Secondly, overexpression of SIRT7 in PCa cells promotes migration and invasion. To date few studies have investigated the expression and the role of SIRT7 in cancer, however, all sirtuins are involved in cancer development through different mechanisms. Generally, sirtuins are considered to be tumor suppressors, but they may also act as tumor promoters particularly in advanced cancers [11–13]. Elevated levels of Sirt7 have been previously observed in several cancers including breast, liver, pancreas and colon [14–17]. SIRT7 is also associated with poor prognosis in gastric, lung, cervical and hepatocellular carcinoma [15, 18–20]. In prostate cancer, the only available data were obtained from the analysis of Cancer Genomics databases of epithelial cancers. In this study, SIRT7 was found elevated in the advanced stages of prostate cancer and Sirt7 amplification was associated with metastasis and poor prognosis [21]. Our immunohistochemical analysis of matched tumor and normal prostate tissue on 57 patients clearly demonstrated that the level of SIRT7 is increased in tumor. Moreover, we showed that high expression of SIRT7 is associated with capsular invasion an indicator of poor prognosis.

Metastasis is a process which requires cancer cell migration and invasion of the adjacent normal tissue. In accordance with a role of SIRT7 in prostate cancer progression, we showed that the knockdown of SIRT7 decreased cancer cell migration but did not alter cell viability. Like our findings, Malik et al. also demonstrated that SIRT7 depletion led to a reduction of PC3 cells migration while it did not interfere with cancer cell proliferation [21]. On contrary, inhibition of SIRT7 has been shown to stop proliferation and trigger apoptosis through the downregulation of the RNA polymerase 1 in NIH-3T3, HEK-293 and U-2 OS cells [22]. Here, we directly associated deacetylase activity of SIRT7 with the aggressiveness of cancer cells since the mutated form of SIRT7, a variant bearing a point mutation in the catalytic site of the protein, did not promote cancer cell migration and invasiveness, unlike the native protein. The upregulation of SIRT7 has been shown to induce ovarian cancer cell migration [23]. Similar observations were done in colorectal cancer cells where the overexpression of SIRT7 increased cell motility and invasiveness associated with an increase of mesenchymal markers [17]. The aggressiveness of the SIRT7 overexpressing cells is consistent with the increase of FN and slug/snail mRNA expression. The increase observed in the cells expressing the mutated protein demonstrates that FN and slug/snail are not sufficient to promote aggressiveness.

Our findings also suggest that the deacetylase activity of SIRT7 is implicated in the resistance to chemotherapy since overexpression of the SIRT7wt but not SIRT7mut induced the resistance of the cells to docetaxel. The role of sirtuins in the resistance to chemotherapy is poorly documented. However, Kiran et al. have demonstrated that the knockdown of SIRT7 renders osteosarcoma cells sensitive to doxorubicin and its overexpression attenuated doxorubicin mediated DNA damage in accordance with our results [24]. In contrast, the activation of SIRT1 has been shown to sensitize breast cancer cells to tamoxifen via FoxO1 and the upregulation of Multidrug Resistance-Associated Protein 2 [25]. In prostate cancer cells SIRT1 inhibition sensitized DU145 to cisplatin. [26]. On the contrary, SIRT1 facilitated chemoresistance in pancreatic cancer cells and the inhibition of SIRT1 sensitizes to gemcitabine [27].

In conclusion, our study demonstrates that SIRT7 is a new promising marker for aggressive prostate cancer. A study including a larger number of patients and a longer follow-up including patient's survival will help to understand the role of SIRT7 in PCa progression.

## MATERIALS AND METHODS

### Immunohistochemistry

We prospectively collected clinical and pathological data from consecutive consenting patients undergoing radical prostatectomy for prostate cancer between 2014 and 2015. Surgical specimens underwent conventional histological processing and examination according to the Stanford protocol. Additionally, in each tissue section, areas of adenocarcinoma were identified and graded according to Gleason Score. Then, the Index lesion corresponding to the highest Gleason Score was studied by immunohistochemistry, using anti-SIRT7 antibody (Prestige Antibodies^®^, SIGMA-ALDRICH^®^). SIRT7 expression was analyzed in a qualitative manner by evaluating positivity or negativity of expression, comparing healthy and tumor areas, and in a quantitative manner. To quantify the expression of SIRT7 we used the Allred Score (0 to 8) [10] obtained by adding proportion of staining tumor (0 to 5) and signal intensity (0 to 3). The quantification was done by determining the average intensity and proportion of nuclei labelled including the nucleolus.

In each index lesion, the Allred Score and Gleason Score were compared using the Student t test (significance with p < 0.05). The analysis was performed blindly by two independent investigators.

### Cell lines and culture conditions

The cell lines were purchased from the ATCC (Manassas, VA, USA). The LNCaP cells were cultured in RPMI 1640 medium, the DU145 and PC3 cells were cultured in DMEM (Invitrogen, Carlsbad, CA, USA) containing 25 mmol/L glucose supplemented with 10% fetal bovine serum (FBS) and 100 units/mL penicillin at 37°C and 5% CO2.

Stable clones expressing SIRT7 were established as follow: cells were transfected with Lipofectamine 2000 (Invitrogen, Carlsbad, CA) with empty vectors, the plasmids expressing wt-SIRT7 or the mutated form of SIRT7 that displays no deacetylase activity (SIRT7 H187Y), a Kind gift of Pr K Chua (Stanford, USA) [9]. Once the clonal population was selected with puromycin (1 μg/ml), cells were characterized to validate the overexpression of SIRT7.

### Cell counting

Cells were seeded in 96 well plates, after the indicated time cells were dissociated with trypsin and counted using Flow cytometry using Dapi to discriminate dead cells from live cells.

### Cell viability assay

Cells were seeded in 96 well-plate and treated with different concentrations of Docetaxel. After 3 days, XTT (Promocell, Germany) was added to the cells and the optical density was measured.

### Cell transfection with siRNA

Cells were transfected with two siRNA targeting different regions of SIRT7: siRNA SIRT7, ID 116146 (Applied biosystem, AMBION, Carlsbad, CA) or ON Target plus siRNA smart Pool (Dharmacon, GE HealthCare, Lafayette, CO) or a negative control siRNA (using Lipofectamine RNAi max (Invitrogen, Carlsbad, CA). After 48 h of transfection, cells were counted and proteins were extracted.

### Colony formation assay

LNCaP cells stably expressing control or various SIRT7 constructs (103 cells/ml) growing in semisolid methyl cellulose medium. MethoCult H4100 was used for cell lines (StemCell Technologies Inc., Vancouver, Canada). Colonies were detected after 10 days of culture by adding 1 mg/ml of 3-(4,5-dimethylthiazol-2-yl)-2,5-diphenyltetrazolium bromide (MTT) reagent and were scored by Image J quantification software (U.S. National Institutes of Health, Bethesda, MD, USA).

### Migration and invasion assays in boyden chambers

Boyden chambers with filter inserts coated with fibronectin (10 μg/ml) and 8-μm pores (BD Bioscience) were used to quantify cell migration. After an overnight serum starvation, 12 × 104 cells were seeded in the upper chamber in serum free medium. The lower chamber contained complete 10% FBS medium. Cell migration was determined after 6h by counting all cells in five randomly selected counting areas at the lower surface of the filter. Cells on the upper surface were removed with a cotton swab; filters were fixed with PFA 4% and incubated with a DAPI solution (2mg/ml) to label cell nuclei. Nuclei were counted and analyzed using Image J software. For invasion experiments, the inserts were coated with 25 μg/μl of Matrigel (BD Bioscience) and cells were counted after 24h.

### Western blotting analysis

Cell extracts were prepared using lysis buffer as described previously [11]. Immunoblotting was performed with antibodies against SIRT7 (Merck-Millipore, Molsheim, France), HSP90 (Santa Cruz Biotechnology, Santa Cruz, CA, USA) and Acetyl Histone H3K18 (Euromedex, France).

### RNA extraction

Total RNA were prepared from the paraffin section with the FFPE RNA kit (Thermo Fisher Scientific, Waltham, MA, USA) following the procedure of the manufacturer.

Total RNA from the cells were extracted with Trizol (Thermo Fisher Scientific, Waltham, MA, USA) as previously described [11].

### Real time PCR

The qPCR was conducted using a Step-one Real Time PCR system with TaqMan Fast Universal Master mix or SYBR Green Master Mix (Applied Biosystems, Life Technologies SAS). For RNA from paraffin section we used the specific TaqMan gene expression assays for human SIRT7 (Hs01034735), AMACR (Hs00204885) and two housekeeping genes: RPLP0 (Hs99999902) and 18s (Hs99999901). For RNA extracted from cells, we used SYBER green based assay, with previously designed oligonucleotides specific for human Fibronectin, Slug/Snail and vimentin (sequence available upon request).

## SUPPLEMENTARY MATERIALS FIGURES


