# Supplementary material for: Sirtuin 7: a new marker of aggressiveness in prostate cancer
Source: Oncotarget. 2017 Aug 24;8(44):77309–16. doi: 10.18632/oncotarget.20468 (PMC5652781; doi:10.18632/oncotarget.20468)
Supplement: Supplementary file 1 [file oncotarget-08-77309-s001.pdf]

Sirtuin 7: a new marker of aggressiveness in prostate cancer

SUPPLEMENTARY MATERIALS

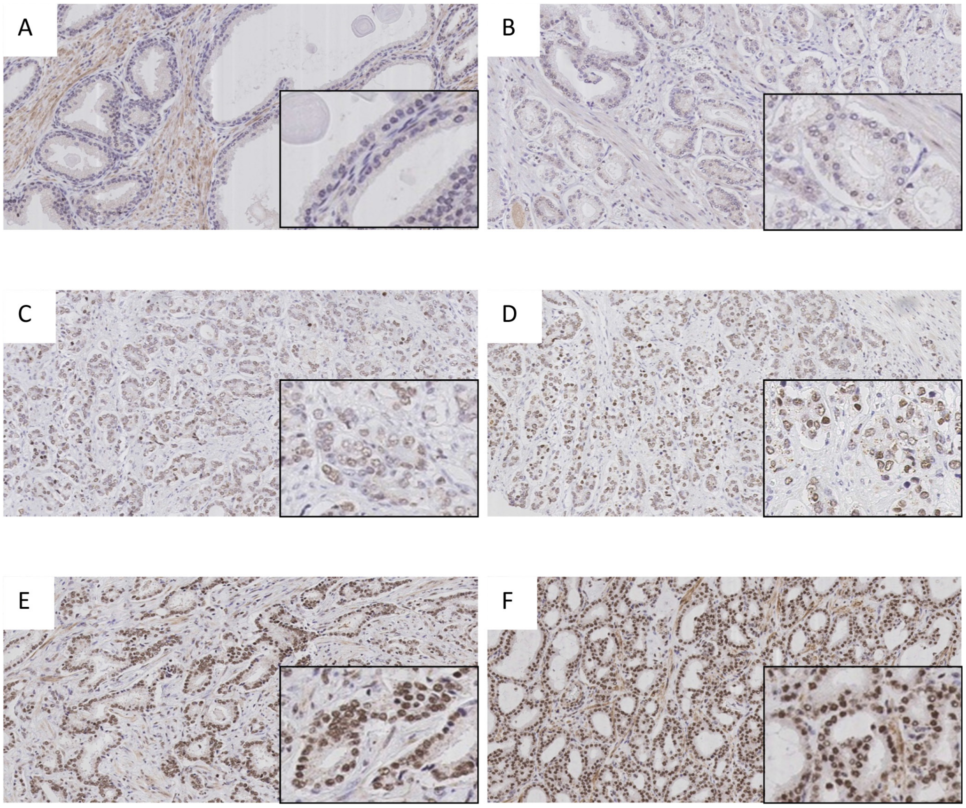

Supplementary Figure 1: Sirtuin 7 immunohistochemical staining on different human prostate tissues. (A) healthy negative; **(B)** tumoral negative; **(C)** tumoral weak; **(D)** tumoral moderate, **(E, F)** tumoral strong. (magnification x200, insert x400).

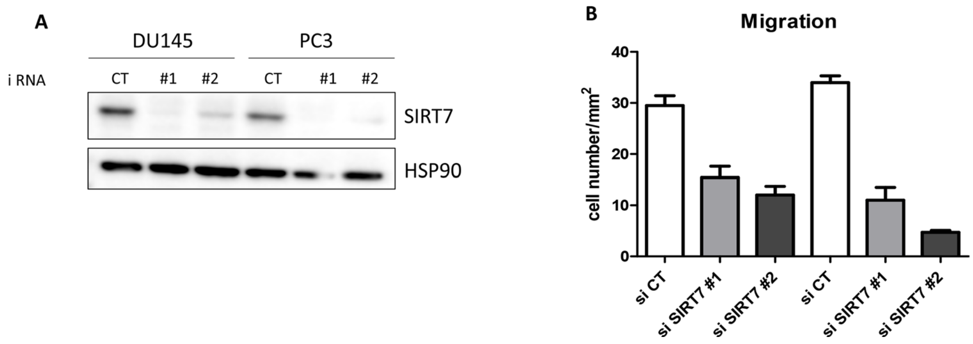

Supplementary Figure 2: The knockdown of SIRT7 by two different siRNA inhibits DU145 and PC3 migration **(A)** Western blotting of SIRT7 in DU145 and PC3 cells transfected with two siRNA targeting different regions of SIRT7. **(B)** The graph represents the migration of the indicated cancer cells as described in the materials and methods.

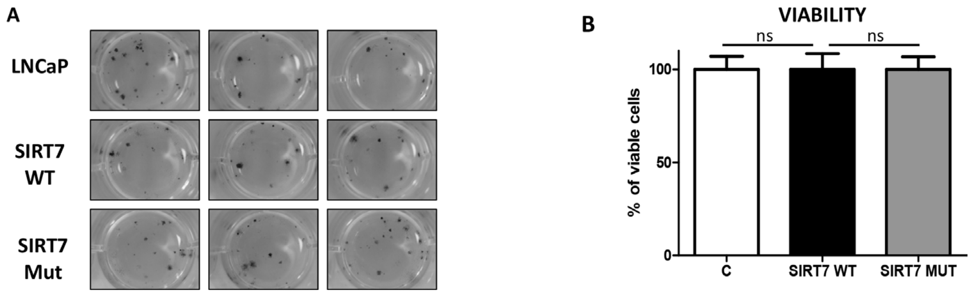

Supplementary Figure 3: SIRT7 overexpression does not affect clonogenic growth and viability. (A) Representative pictures of a clonogenic assay with LNCaP cells, cells expressing SIRT7wt and SIRT7mut. **(B)** Cell viability of control cells, cells expressing SIRT7wt and SIRT7mut. The data represents three independent experiments with sem.
